# Composition of Total and Cell-Proliferating Bacterioplankton Community in Early Summer in the North Sea – Roseobacters Are the Most Active Component

**DOI:** 10.3389/fmicb.2017.01771

**Published:** 2017-09-13

**Authors:** Insa Bakenhus, Leon Dlugosch, Sara Billerbeck, Helge-Ansgar Giebel, Felix Milke, Meinhard Simon

**Affiliations:** Institute for Chemistry and Biology of the Marine Environment, University of Oldenburg Oldenburg, Germany

**Keywords:** bacteria, community composition, CARD-FISH, BrdU-FISH, Roseobacter, North Sea

## Abstract

Heterotrophic bacterioplankton communities play an important role in organic matter processing in the oceans worldwide. In order to investigate the significance of distinct phylogenetic bacterial groups it is not only important to assess their quantitative abundance but also their growth dynamics in relation to the entire bacterioplankton. Therefore bacterial abundance, biomass production and the composition of the entire and cell-proliferating bacterioplankton community were assessed in North Sea surface waters between the German Bight and 58°N in early summer by applying catalyzed reporter deposition (CARD-FISH) and bromodeoxyuridine fluorescence *in situ* hybridization (BrdU-FISH). *Bacteroidetes* and the *Roseobacter* group dominated the cell-proliferating fraction with 10–55 and 8–31% of total BrdU-positive cells, respectively. While *Bacteroidetes* also showed high abundances in the total bacterial fraction, roseobacters constituted only 1–9% of all cells. Despite abundances of up to 55% of total bacterial cells, the SAR11 clade constituted <6% of BrdU-positive cells. *Gammaproteobacteria* accounted for 2–16% of the total and 2–13% of the cell-proliferating cells. Within the two most active groups, BrdU-positive cells made up 28% of *Bacteroidetes* as an overall mean and 36% of roseobacters. Estimated mean growth rates of *Bacteroidetes* and the *Roseobacter* group were 1.2 and 1.5 day^-1^, respectively, and much higher than bulk growth rates of the bacterioplankton whereas those of the SAR11 clade and *Gammaproteobacteria* were 0.04 and 0.21 day^-1^, respectively, and much lower than bulk growth rates. Only numbers of total and cell-proliferating roseobacters but not those of *Bacteroidetes* and the other groups were significantly correlated to chlorophyll fluorescence and bacterioplankton biomass production. The *Roseobacter* group, besides *Bacteroidetes*, appeared to be a major player in processing phytoplankton derived organic matter despite its low partitioning in the total bacterioplankton community.

## Introduction

Heterotrophic bacterioplankton communities play an important role in the cycling of carbon, nitrogen and other nutrients in the oceans worldwide. About half of the phytoplankton primary production, supplied in the form of dissolved (DOM) and particulate organic matter (POM), is degraded and mineralized by heterotrophic bacteria (Azam and Malfatti, 2007). The metabolic pathways to break down the complex DOM and POM are diverse and carried out by a multitude of members of the bacterioplankton, distinct in their growth, substrate and environmental requirements (Teeling et al., 2012; Lucas et al., 2015; Sunagawa et al., 2015; Milici et al., 2016; Osterholz et al., 2016). Over the last decade, our insight into the community structure and functional diversity of the bacterioplankton has improved greatly with the establishment of culture-independent methods and in particular the application of fluorescence *in situ* hybridization (FISH) and next generation sequencing technologies. In oceanic environments, *Alpha-* and *Gammaproteobacteria* as well as *Flavobacteria* and *Sphingobacteria* of the *Bacteroidetes* phylum constitute the major fractions of bacterioplankton communities but other phylogenetic lineages, such as *Actinobacteria* and *Planctomycetes*, contribute as well (Schattenhofer et al., 2009; Wietz et al., 2010; Teeling et al., 2012; Buchan et al., 2014; Sunagawa et al., 2015; Milici et al., 2016). Even though the quantitative contribution to the community provides a first view into the significance of a given bacterial taxonomic group, it does not provide clear-cut information on its functional significance and role in organic matter processing. This type of information may be provided by assessing the composition of the metabolically active members, relative to the total bacterioplankton community on the basis of (1) the 16S rRNA and its gene or its expression patterns (Campbell et al., 2011; Gifford et al., 2014), (2) metatranscriptomic analyses (Ottesen et al., 2011; Varaljay et al., 2015), (3) FISH-based activity measurements coupled to microautoradiography (MAR-FISH; Cottrell and Kirchman, 2000; Malmstrom et al., 2007) or finally (4) bromodeoxyuridine (BrdU-FISH; Pernthaler et al., 2002b; Tada et al., 2011). Whereas the former provide detailed information on expression patterns of functional genes with a high phylogenetic resolution only MAR-FISH and BrdU-FISH yield quantitative data on the numeric abundance of metabolically active, i.e., protein synthesizing or DNA proliferating bacterial taxa. Whereas MAR-FISH applies tritiated substrates including leucine and thymidine to trace protein synthesizing and DNA proliferating cells BrdU-FISH applies a thymidine analog for tracing DNA proliferating cells. A compilation of many studies showed that on average 40% of total cells are detected as metabolically active by MAR-FISH (del Giorgio and Gasol, 2008). Similar data are not yet available for BrdU-FISH as only few studies applied this approach (Pernthaler et al., 2002b; Tada et al., 2011, 2013). Proportions of BrdU-active cells in these studies range between 5 and 37%.

In a finer scale the active fraction of a given phylogenetic bacterial group may vary greatly, from <10% to >40% as demonstrated by studies in the Arctic and Atlantic Ocean (Malmstrom et al., 2007; Alonso-Sáez et al., 2012), the Southern Ocean (Straza et al., 2010; Tada et al., 2013) and the western North Pacific (Tada et al., 2011). *Bacteroidetes* and SAR11, often quantitatively dominating bacterioplankton communities, usually constitute only relatively low fractions of the active cells, whereas the *Roseobacter* group, constituting much lower fractions of bacterioplankton communities, often exhibit high fractions, i.e., >50%, of active cells (Malmstrom et al., 2007; Tada et al., 2011, 2013; Alonso-Sáez et al., 2012). While this observation was in particular oceanic environments, it may suggest more broadly, that the *Roseobacter* group contributes relatively more than the other bacterial groups to organic matter processing but is more susceptible to mortality including grazing and viral lysis.

The North Sea is a coastal sea with pronounced on-off shore gradients of inorganic nutrients, DOM, phytoplankton biomass and bacterioplankton growth and community composition (McQuatters-Gollop et al., 2007; Giebel et al., 2011; Osterholz et al., 2016). Bacterioplankton community dynamics have recently been studied extensively in the German Bight of the North Sea (Teeling et al., 2012; Wemheuer et al., 2014; Lucas et al., 2015; Voget et al., 2015). Despite these extensive studies still little is known about the relative and absolute significance of these phylogenetic groups for bacterial biomass production and cell proliferation. Therefore the aim of this study was to assess cell proliferation of these bacterial groups by BrdU-FISH together with bacterial biomass production and relevant microbial and geochemical variables in early summer between the German Bight and the northern North Sea at 58°N.

## Materials and Methods

### Study Area and Sampling

**FIGURE 1:**
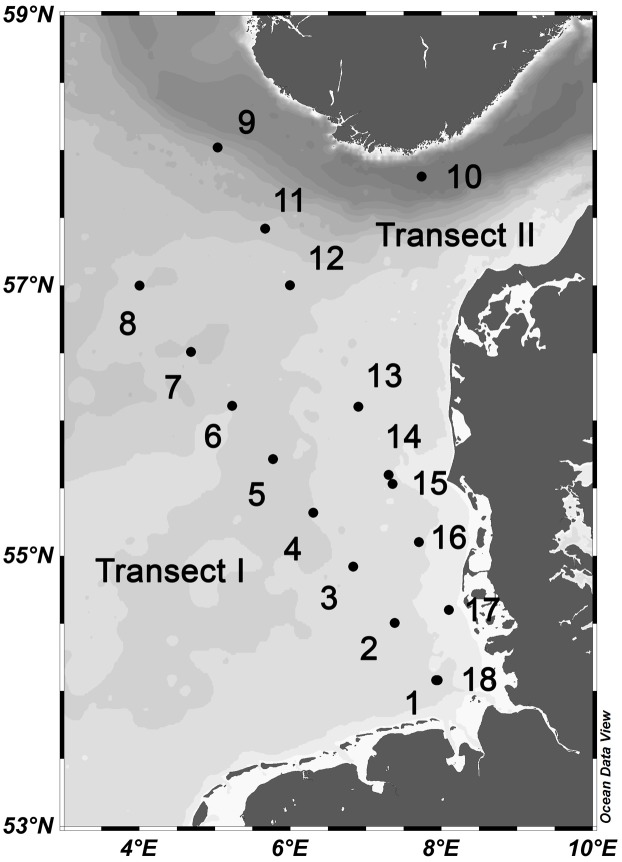
Map of the study area in the North Sea with station numbers of Transect I (1–8) and II (9–18). For exact location of the stations see Supplementary Table S1.

Surface samples were collected during a cruise with RV Heincke in the North Sea at 3 m depth between the German Bight at ∼54°N and 58°N west of Norway from 23 May to 7 June 2014 (**Figure 1** and Supplementary Table S1). On the way north a more westerly transect with eight stations (Transect I) was sampled from 23 to 26 May whereas on the way south a more easterly and coastal transect (Transect II) with nine stations and an additional station in the Norwegian Trench (station 10) was visited between 27 May and 5 June. Depth profiles of conductivity, temperature and chlorophyll *a* (Chl*a*) fluorescence were measured at each station using a CTD sensor (OTS 1500, Meereselektronik, Kiel, Germany) and a Wetlabs ECO FL fluorometer (Philomath, OR, United States). Samples were collected by 5-L Niskin bottles mounted on a rosette (Hydrobios, Kiel, Germany). Subsamples were withdrawn from the bottles immediately after retrieval and further processed for various measurements (see below).

### Biogeochemical and Microbial Variables

Subsamples for the analysis of particulate organic carbon (POC) and nitrogen (PON) were filtered onto precombusted (2 h, 450°C) and preweighed GF/F filters (Whatman). Filters were rinsed with distilled water to remove salt and kept frozen at -20°C until analysis as described in Lunau et al. (2006). Subsamples for Chl*a* and phaeopigments were filtered onto GF/F filters (Whatman, 47 mm diameter), immediately wrapped into aluminum foil and kept frozen at -20°C until further spectrophotometric analysis in the lab. The spectrophotometric analysis was performed as described in Giebel et al. (2011). Numbers of prokaryotic cells, referred as bacterioplankton hereafter, were determined by flow cytometry as described in Osterholz et al. (2015). The cells of the bacterioplankton community were discriminated in high nucleic acid (HNA) and low nucleic acid (LNA) content cells by their distinct fluorescence yield and each subpopulation was delineated via manual gating in a plot of green fluorescence (FL1, 533 ± 15 nm) vs. red fluorescence (FL3, >670 nm).

Rates of bacterioplankton biomass production (BP) were determined by the incorporation of ^14^C-leucine. Briefly, triplicate 5-mL subsamples and a formalin-killed control were incubated with ^14^C-leucine (10.8 GBq mmol^-1^, Hartmann Analytic, Germany) at a final concentration of 20 nM in the dark at *in situ* temperature for 1 h and further processed as described (Lunau et al., 2006). Biomass production was calculated using a conversation factor of 3.05 kg C (mol leucine)^-1^ according to Simon and Azam (1989).

Bacterioplankton community growth rates (μ; day^-1^) were calculated as μ = ln(B1)-ln(B0), where B0 and B1 (B0+BP) are bacterioplankton biomass at T0 and 1 h later. Bacterioplankton biomass was calculated from bacterial cell numbers, assuming a carbon content of 20 × 10^-15^ g C per cell (Simon and Azam, 1989) and BP is bacterioplankton biomass production as outlined above.

### CARD- and BrdU-FISH

Seawater samples of 55 mL were transferred to dark bottles and incubated with 5-bromo-2′-deoxyuridine (BrdU) (Sigma–Aldrich, Germany; 20 μM final concentration) and thymidine (Sigma–Aldrich, Germany; 33 nM final concentration). A formaldehyde-fixed sample (2% final concentration) served as control. After 4 h incubation at *in situ* temperature samples were fixed with formaldehyde at a final concentration of 2% (v/v) for 1 h at room temperature, filtered onto 0.2 μm polycarbonate filters (Whatman) and stored at -20°C until further analysis in the lab.

Bacterioplankton community composition was analyzed by catalyzed reporter deposition fluorescence *in situ* hybridization (CARD-FISH) using horseradish peroxidase-labeled oligonucleotides probes specific for *Bacteroidetes*, the *Bacteroidetes* subgroup *Polaribacter*, *Gammaproteobacteria*, the SAR11 clade, the *Roseobacter* group and the *Roseobacter* subgroup RCA (*Roseobacter* clade affiliated) cluster (**Table 1**). Analysis was carried out according to Pernthaler et al. (2002a), but hybridization and amplification in glass humidity chambers according to Bennke et al. (2013) with a hybridization time of 2 h at 46°C. Hybridization with the probe SAR11-441R was carried out using a 45% [vol/vol] formamide hybridization buffer at 35°C overnight. Oligonucleotide probes as well as unlabeled competitor (Manz et al., 1992) and helper probes (Fuchs et al., 2000) targeting the 16S rRNA of the RCA cluster were newly designed by evaluating probes for the RCA cluster with increased group coverage compared to probe RCA826 (Selje et al., 2004) by using the probe design tool in ARB (Ludwig et al., 2004). Analyses of hybridization efficiency via mathFISH^1^ (Supplementary Table S2; Yilmaz et al., 2011) as well as hybridization with RCA isolate RCA23 (*Planktomarina temperata*, GenBank Accession Number GQ369962, Supplementary Figure S1) resulted in best efficiency and specificity for probe RCA996 covering 91% of the sequences of the RCA cluster (Silva SSU Ref NR 128 database, Supplementary Figure S2). For determining optimal stringency conditions of probe RCA996 a series of hybridizations at formamide concentrations from 0 to 70% were carried out against two RCA isolates, RCA23 and LE17 (*Roseobacter* sp., GenBank Accession Number GQ468665). A *Paracoccus* strain (*Paracoccus* sp. GWS-BW-H72M, GenBank Accession Number AY515424) served as negative control. Optimal formamide concentration of 35% was defined as the highest concentration before decreasing signal intensity.

**Table 1:** Probes, their target group, sequence data and formamide (FA) concentration used for this study.

Probe	Target group	Sequence 5′–3′	FA (%)	Reference
CF319a	*Bacteroidetes*	TGG TCC GTGTCT CAG TAC	35	Manz et al., 1996
POL740	*Polaribacter*	CCC TCA GCG TCA GTA CAT ACG T	35	Malmstrom et al., 2007
ROS536	*Roseobacter*	CAA CGC TAA CCC CCT CCG	35	Brinkmeyer et al., 2000
	Competitor	CAA CGC TAG CCC CCT CAG		
RCA996	RCA cluster	TCT CTG GTA GTA GCA CAG GAT	35	This study
	Competitor	CTC TGG GAG TAG CAC AGG		
	Helper978	GTC AAG GGT TGG TAA GGT		
	Helper1017	CCC GAA GGG AAC GTA CCA		
Gam42a	*Gammaproteobacteria*	GCC TTC CCA CAT CGT TT	35	Manz et al., 1992
	Competitor Bet42a	GCC TTC CCA CTT CGT TT		
SAR11-441R	SAR11-clade	TAC AGT CAT TTT CTT CCC CGA C	45	Morris et al., 2002
NON338	Negative control	ACT CCT ACG GGA GGC AGC	35	Wallner et al., 1993


In the results, proportions of the *Roseobacter* group and of *Bacteroidetes* are presented as those of the total minus the RCA cluster and minus the *Polaribacter* cluster, respectively. The latter subgroups are given as separate data such that the total of *Roseobacter*+RCA and *Bacteroidetes*+*Polaribacter* represent proportions of the total *Roseobacter* group and the *Bacteroidetes* phylum.

BrdU-FISH was performed according to Pernthaler et al. (2002b) but with the following modifications: The enzymatic permeabilization time was extended to 45 min. The digestion of intercellular DNA during the antibody-reaction was carried out separately by incubation of the filter sections for 30 min at 37°C in digestion buffer (50 mM Tris-HCl, 5 mM MgCl_2_) and using the restriction enzymes ExoIII (53.3 U/mL) and HaeIII (20 U/mL). The antibody reaction was shortened to 2 h and the washing step after the BrdU-amplification carried out in 1x PBS buffer. Proportions of the *Roseobacter*+RCA group and the *Bacteroidetes*+*Polaribacter* group are shown in the same way as the CARD-FISH data (see above).

Microscopic images of the filter sections were acquired semi-automatically using the epifluorescence microscope AxioImager.Z2m including the software package AxioVisionVs 40 V4.8.2.0 (Carl Zeiss, Jena, Germany). Relative abundances of the phylogenetic groups were determined using the automated image analysis software ACMEtool3 (©M. Zeder^2^). Absolute numbers of bacteria detected by CARD-FISH and BrdU-FISH, respectively, were calculated from the relative abundances determined by CARD-FISH and BrdU-FISH, respectively, and the total bacterial numbers obtained by flow cytometry. Negative control counts (hybridization with HRP-Non338) were always below 1% of DAPI-stained cells.

### Statistical Analysis

Pearson’s correlation analysis (95% confidence interval) was performed to determine the correlation between the abundance of CARD-FISH targeted and BrdU-positive bacterial groups and environmental, microbial and biogeochemical variables.

## Results

### Environmental and Biogeochemical Characteristics

Water temperature ranged from 11 to 16°C with lower values in the northern North Sea (Supplementary Table S1). Salinity varied between 24.5 and 35.1 with the lowest salinities at stations 9 and 10 close to the Norwegian coast affected by Baltic Sea water (Supplementary Table S1). Salinities in the German Bight ranged between 29.9 and 32.8 and further north and offshore between 33.0 and 35.1.

**FIGURE 2:**
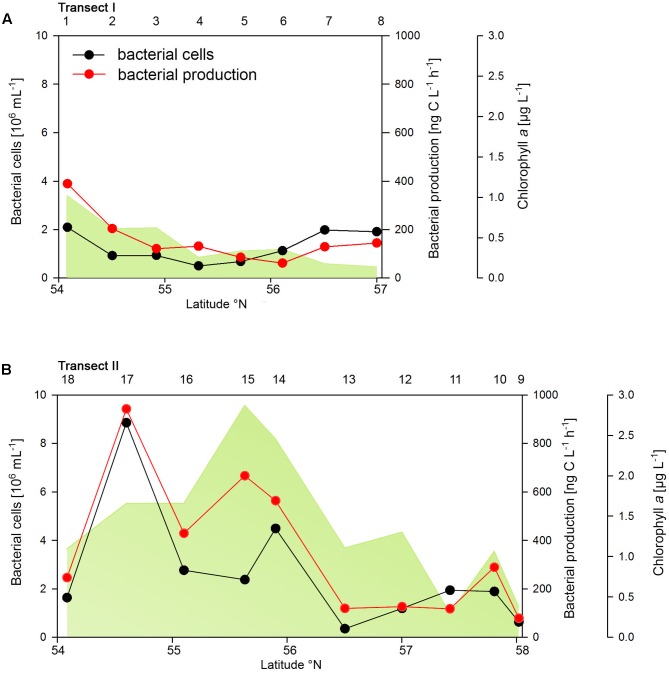
Bacterial cell numbers, biomass production and chlorophyll *a* concentrations measured spectrophotometrically of Transects I **(A)** and II **(B)** during early summer in the North Sea between 54° and 58°N. The numbers above the panels indicate station numbers.

The two transects differed markedly in their biogeochemical and microbial properties. Transect I, located in the more off shore westerly region, exhibited generally lower concentrations of POC, PON, Chl*a* and bacterioplankton cell numbers and rates of BP than Transect II which stretched over the easterly coastal regions (**Figure 2** and Supplementary Table S1). On Transect I Chl*a* concentrations did not exceed 1.0 μg L^-1^ and gradually decreased toward the northern region to <0.2 μg Chl*a* L^-1^ (**Figure 2A**). In contrast, on Transect II concentrations exceeded 1.0 μg Chl*a* L^-1^ at 8 of the 10 stations with maxima of 2.5 and 2.8 μg Chl*a* L^-1^ between 55° and 56°N (**Figure 2B**). POC and PON also exhibited higher concentrations on Transect II than on Transect I and covaried with Chl*a* (Supplementary Table S1).

### Bacterioplankton Abundance and Growth

**FIGURE 3:**
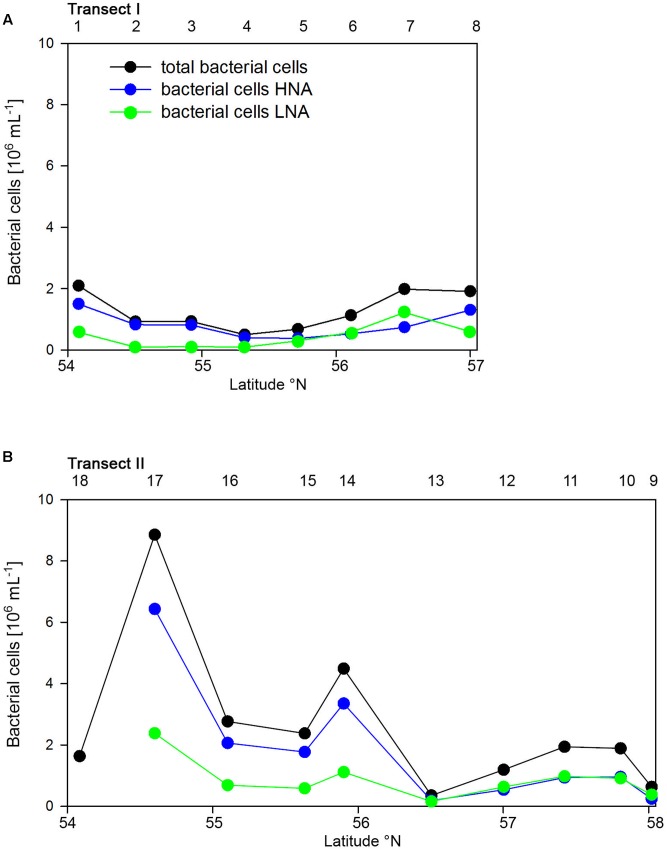
Numbers of total, high (HNA) and low nucleic acids (LNA) containing bacterial cell of Transects I **(A)** and II **(B)** during early summer in the North Sea between 54° and 58°N. The numbers above the panels indicate station numbers.

**FIGURE 4:**
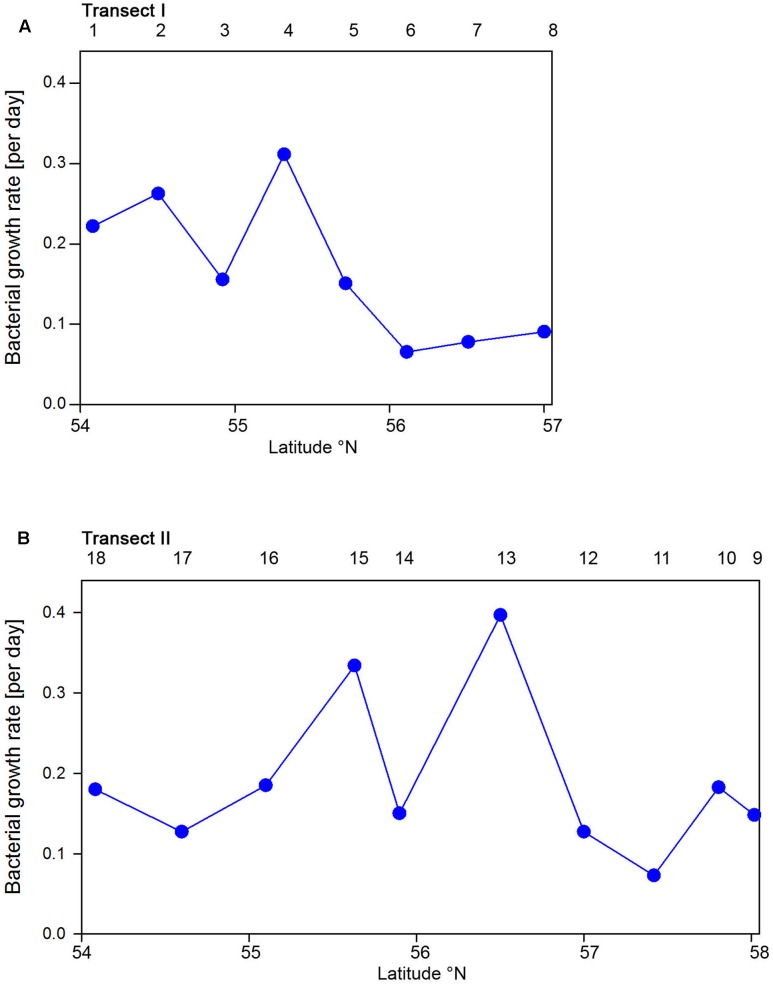
Bacterioplankton bulk growth rates of Transects I **(A)** and II **(B)** during early summer in the North Sea between 54° and 58°N. The numbers above the panels indicate station numbers.

Bacterioplankton cell numbers on Transect I ranged between 0.5 × 10^6^ and 2.1 × 10^6^ mL^-1^ with lowest values in the central part of the transect (**Figure 2A**). At the four southern stations at least 72% of the cells were HNA cells whereas at stations 5 to 7 the fraction of HNA cells was reduced to between 55 and 37% (**Figure 3A**). On Transect II bacterioplankton abundance varied greatly with highest cell numbers of 8.9 × 10^6^ and 4.5 × 10^6^ mL^-1^ at 54.60° and 55.60°N, respectively, and values of <2.0 × 10^6^ mL^-1^ at stations further north and at the southernmost station (**Figure 2B**). South of 56°N HNA cells dominated by 73–75% whereas at 56.50°N and further north HNA constituted only 39–54% (**Figure 3B**). Bacterioplankton biomass production generally covaried with bacterioplankton cell numbers with rates not exceeding 400 ng C L^-1^ h^-1^ on Transect I, gradually decreasing toward the northern region, and rates of up to 942 ng C L^-1^ h^-1^ in the southern part of Transect II (**Figure 2**). Both variables in the entire data set were highly significantly linearly correlated (*r*^2^ = 0.77, *p* < 0.001). Bacterioplankton community growth rates ranged between 0.06 and 0.4 per day (**Figure 4**). Highest values occurred on Transect I at stations 2 and 4 and on Transect II at stations 13 and 15. Growth rates did not covary with other microbial or biogeochemical variables.

### Bacterioplankton Community Structure

**FIGURE 5:**
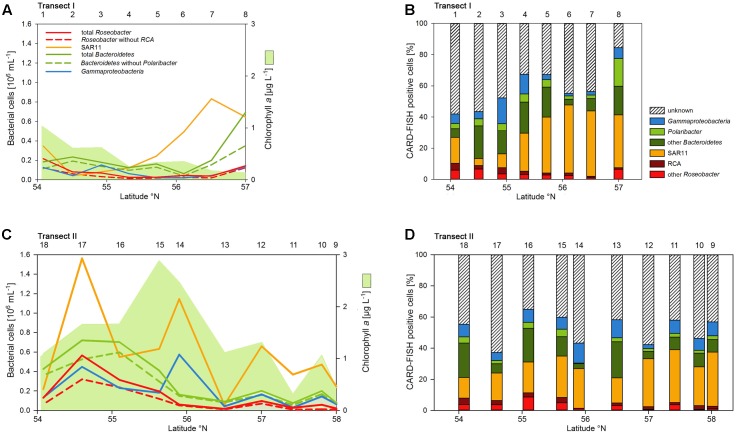
Cell numbers of the SAR11 clade, the *Roseobacter* group and its RCA cluster, *Bacteroidetes* and its *Polaribacter* cluster and of *Gammaproteobacteria* assessed by CARD-FISH and chlorophyll *a* concentrations measured spectrophotometrically of Transects I (upper panel) and II (lower panel) during early summer in the North Sea between 54° and 58°N. The red bars represent the *Roseobacter* group without the RCA cluster and the dark green bars *Bacteroidetes* without the *Polaribacter* cluster. The numbers above the panels indicate station numbers. **(A,C)** Show absolute cell numbers and chlorophyll *a* and **(B,D)** percentages of DAPI-stained cells detected by a group-specific CARD-FISH probe.

Abundances of the bacterioplankton phylogenetic groups detected by CARD-FISH generally covaried with total bacterioplankton cells as shown by highly significant linear correlations of all phylogenetic groups (mean *r*^2^ = 0.73, range 0.62–0.90; *p* < 0.001) except the *Polaribacter* cluster (**Figure 5**). On Transect I 42 to 84% of total cells were identified by the probes applied and on Transect II 37 to 64% (**Figures 5A,C**). SAR11 was the most abundant group and dominated the bacterioplankton communities except at three stations on Transects I and II, respectively (**Figures 5A,C**). This group constituted 4.5 to 43.8% of total bacterioplankton cells on Transect I and 13.4 to 34.8% on Transect II (**Figures 5B,D**). The second most abundant group was *Bacteroidetes* including the *Polaribacter* cluster, which comprised 5.7 to 36.2% on Transect I and 3.6 to 25.9% on Transect II (**Figures 5B,D**). Eight to 49% of this phylogenetic group belonged to the *Polaribacter* cluster (**Figures 5B,D**). *Gammaproteobacteria* constituted 1.9 to 16.4% and 2.5 to 12.8% of total bacterioplankton cells on Transects I and II, respectively (**Figures 5B,D**) and the *Roseobacter* group 2 to 10.4% and 1.3 to 11.3% on Transects I and II, respectively. The *Roseobacter* RCA cluster accounted for 14 to 80% of this group (**Figures 5B,D**).

**FIGURE 6:**
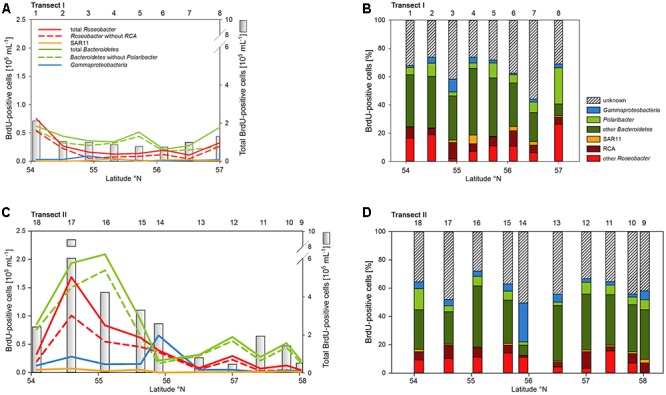
Cell numbers of the SAR11 clade, the *Roseobacter* group and its RCA cluster, *Bacteroidetes* and its *Polaribacter* cluster and of *Gammaproteobacteria* assessed by BrdU-FISH and numbers of total BrdU-positive cells of Transects I (upper panel) and II (lower panel) during early summer in the North Sea between 54° and 58°N. The red bars represent the *Roseobacter* group without the RCA cluster and the dark green bars *Bacteroidetes* without the *Polaribacter* cluster. The numbers above the panels indicate station numbers. **(A,C)** Show absolute cell numbers and **(B,D)** percentages of CARD-FISH cells detected by a group-specific BrdU-FISH probe.

Numbers of DNA-proliferating, i.e., BrdU-positive cells exhibited generally similar patterns as total bacterioplankton cell numbers on both transects (**Figures 6A,C**). Their proportion ranged from 5 to 22% of total cells with an overall mean of 11%. Forty four to 75% (mean = 62 ± 9%) of total BrdU-positive cells could be assigned to cells detected by CARD-FISH and therefore to a bacterial phylogenetic group.

**FIGURE 7:**
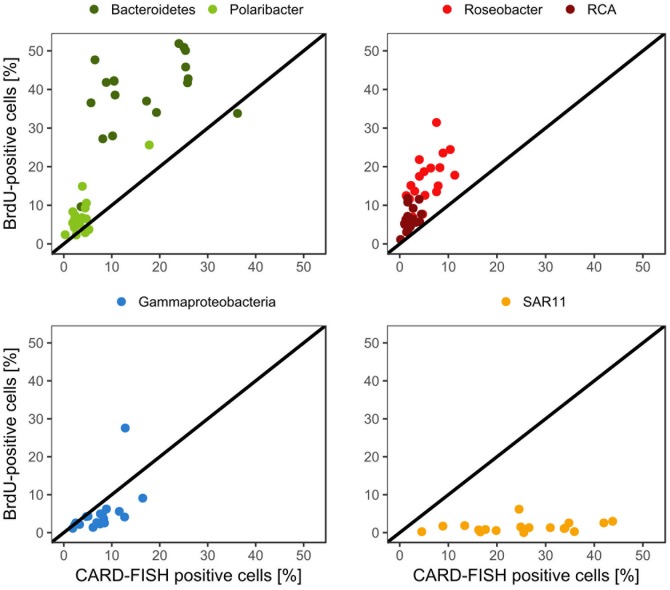
Relative abundances of the *Roseobacter* group and its RCA cluster, *Bacteroidetes* and its *Polaribacter* cluster, the SAR11 clade and of *Gammaproteobacteria* assessed by CARD-FISH and BrdU-FISH during early summer in the North Sea between 54° and 58°N. The red dots represent the *Roseobacter* group without the RCA cluster and the dark green dots *Bacteroidetes* without the *Polaribacter* cluster. The diagonal line indicates equal percentages of the cells assessed by both methods.

*Bacteroidetes* and the *Roseobacter* group dominated the DNA-proliferating cells at all except two stations and constituted 10–52% (mean = 39 ± 10%) and 8–31% (mean = 17±6%) of total BrdU-positive cells, respectively (**Figures 6**, **7**). The SAR11 clade always contributed <6% and as an overall mean only 1.5% to the DNA-proliferating cells (**Figures 6B,D**). Also *Gammaproteobacteria* exhibited low proportions of DNA-proliferating cells with an overall mean of 5%. Only at station 14 on Transect II this group constituted a larger fraction (28%; **Figure 6D**). The *Roseobacter* group with its RCA cluster and *Bacteroidetes* with its *Polaribacter* cluster were clearly overrepresented among the BrdU-positive cells whereas SAR11 and *Gammaproteobacteria* were greatly underrepresented among those cells (**Figure 7**).

**FIGURE 8:**
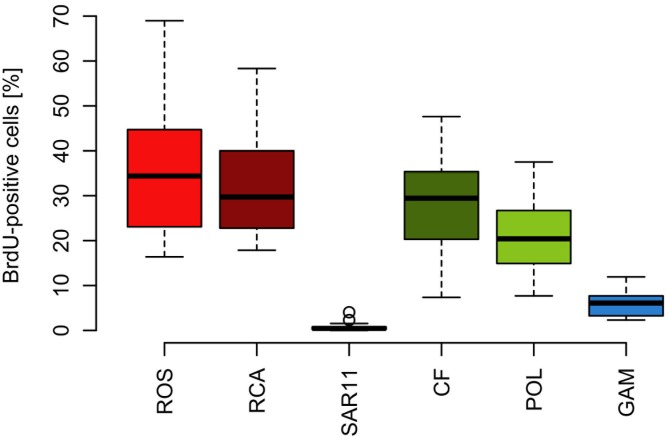
Box-Whisker plots of the percentage of BrdU-positive cells within the *Roseobacter* group and its RCA cluster, the SAR11 clade, *Bacteroidetes* (CF) and its *Polaribacter* cluster and *Gammaproteobacteria* assessed by CARD-FISH during early summer in the North Sea between 54° and 58°N. Given are means, the 25 and 75% percentiles and the error bars of 5 and 95%. The two circles of the SAR11 clade are values above the 95% percentile.

The proportion of DNA-proliferating cells within each phylogenetic group reflects the relative cell proliferation and thus growth activity of the respective group (Pernthaler et al., 2002b). Generally this feature was in line with the proportion of the given group of total BrdU-positive cells (**Figures 7**, **8**). Within *Bacteroidetes* 28% were BrdU-positive as an overall mean and 21% within its subcluster *Polaribacter* (**Figure 8**). The *Roseobacter* group harbored even higher proportions of BrdU-positive cells with a mean of 36 and 32% of its RCA cluster (**Figure 8**). In contrast, within the SAR11 clade <4% and as a mean 1% of the cells were BrdU-positive. Similarly, on average, only 5% of *Gammaproteobacteria* were BrdU-positive (**Figure 8**).

### Correlation Analysis

**FIGURE 9:**
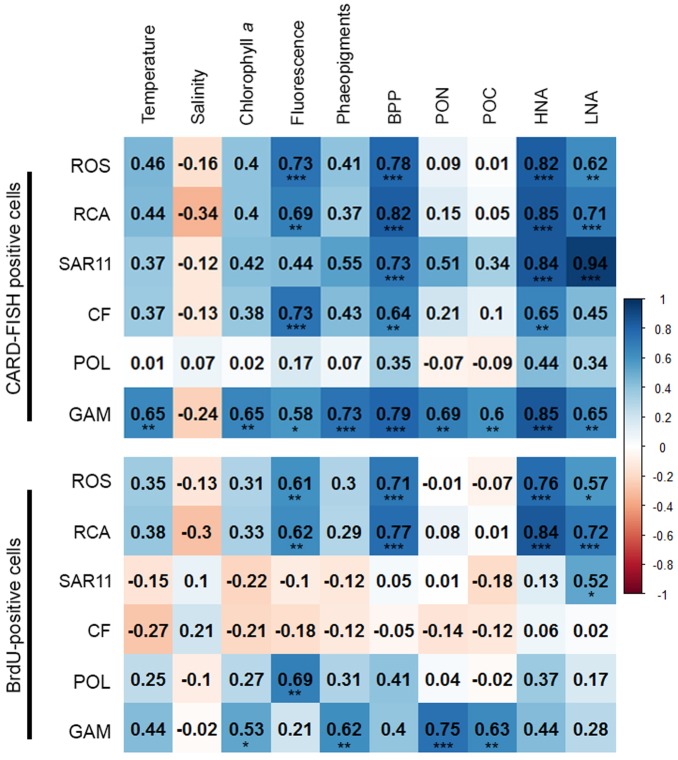
Matrix of the Pearson correlation coefficients (*r*) between absolute numbers of the various bacterial groups assessed by CARD-FISH **(upper)** and BrdU-FISH **(lower)** and hydrographic, microbial and biogeochemical variable assessed during early summer in the North Sea between 54° and 58°N. Color code: blue – positive correlations; red – negative correlations. ^∗^*p* < 0.05, ^∗∗^*p* < 0.01, ^∗∗∗^*p* < 0.001.

In order to obtain a more refined insight into possible factors controlling the dynamics of the different bacterial groups we carried out a Pearson correlation analysis of their absolute numbers based on the CARD- and BrdU-FISH data vs. the assessed hydrographic, biogeochemical and microbial variables. Regarding the CARD-FISH data *Gammaproteobacteria* exhibited highly significant (*p* < 0.001) correlations with nine of the 10 variables tested (**Figure 9**). Correlations with an *r* > 0.7 existed for phaeopigment concentrations, BP and HNA cells. The *Roseobacter* group and its RCA cluster were highly significantly correlated with concentrations of Chl*a*, BP, HNA, and LNA cells and r was larger than 0.7 in three of the four correlations. SAR11 was highly significantly correlated to BP and HNA and LNA cells with an *r* = 0.94 for the latter. *Bacteroidetes* were highly significantly correlated to Chl*a* fluorescence, BP and HNA cells.

The correlation analysis of the absolute numbers of BrdU-positive cells with the same variables yielded quite different results (**Figure 9**). *Gammaproteobacteria* were also significantly (*p* < 0.01) correlated to concentrations of Chl*a* and highly significantly (*p* < 0.001) to phaeopigments, POC and PON but not to the other variables of the correlation of the CARD-FISH data. The BrdU-positive cells of the *Roseobacter* group and its RCA cluster exhibited rather similar highly significant correlations as the CARD-FISH data of this group with the strongest correlations to BP and HNA cells. *Bacteroidetes* did not exhibit any significant correlation and SAR11 only one to LNA cells.

## Discussion

The combined analysis of the bacterioplankton communities by CARD-FISH, BrdU-FISH and by their biomass production on the background of relevant biogeochemical and phytoplankton-related variables allowed us to obtain a rather detailed insight into the growth dynamics of the major bacterioplankton groups and how they were involved in organic matter processing in the North Sea in early summer. On the two transects we encountered different situations which reflected the trophic states of the coastal, nutrient richer, and the off shore, more oligotrophic region of the North Sea (McQuatters-Gollop et al., 2007). Transect I in the more oligotrophic region exhibited generally lower concentrations of POC, Chl*a*, bacterioplankton numbers and BP than Transect II in the more coastal region with particularly high values in the southern part. As bacterioplankton abundance and biomass production closely covaried and community bulk growth rates did not show pronounced differences between both transects this indicates that the general growth control, mainly by substrate supply and losses by grazing and viral lysis (Longnecker et al., 2010; Winter et al., 2010; Chow et al., 2014), was fairly similar.

The SAR11 clade dominated the bacterioplankton community at the great majority of the stations and *Bacteroidetes* was the second most abundant phylogenetic group, even dominating at a few stations on both transects in the southern part. *Gammaproteobacteria* and the *Roseobacter* group constituted lower proportions than the former two groups. This partitioning of the various phylogenetic groups in structuring the bacterioplankton communities is in line with many previous reports from the North Sea and other marine pelagic systems in the temperate zone (Schattenhofer et al., 2009; Tada et al., 2011, 2013; Teeling et al., 2012; Wemheuer et al., 2014; Lucas et al., 2015). The fact that *Bacteroidetes*, the *Roseobacter* group and *Gammaproteobacteria* exhibited close correlations with Chl*a* fluorescence, BP, HNA and LNA cells suggests that their abundance and growth dynamics were controlled by phytoplankton biomass-related dynamics. As *Gammaproteobacteria* exhibited also close correlations to temperature, PON and POC this phylogenetic subclass appeared to respond to dynamics of non-phytoplankton related biogeochemical variables as well. In contrast, the SAR11 clade was correlated only to BP and HNA and LNA cells, obviously reflecting its general dominance in the bacterioplankton community without a distinct response to phytoplankton-related variables.

Our CARD-FISH analysis detected between 37 and 84% of total bacterial cells and as an overall mean 50% leaving room for undetected phylogenetic groups or bacterial cells. It must be kept in mind that the probes we applied may not have covered all phylogenetic bacterial groups known to be present in the North Sea such as the SAR86 and SAR116 clades (Teeling et al., 2012; Wemheuer et al., 2014; Lucas et al., 2015). On the other hand it may also be a result of methodological constraints of our CARD-FISH analysis by small cells below the cut off of our image analysis system.

The BrdU-positive cells constituted as a mean 11% of total bacterioplankton cells. This percentages is in the same range as reported from a study carried out between subtropical and Antarctic waters (Tada et al., 2013) and somewhat lower than reported from the western North Pacific (Tada et al., 2011). Since during the incubation assay BrdU accumulates in the cells over time the proportion of BrdU-positive cells is a function of the incubation time and yields information on the relative cell-proliferation rate of the various phylogenetic groups. We chose an incubation time of 4 h whereas in the two studies cited bacterioplankton communities were incubated for 10 h. This implies that in our study the proportions of the BrdU-positive cells presumably were underestimated as compared to the two other studies. A longer incubation time may not have reached proportions of active bacterial cells as high as by MAR-FISH (∼40%; del Giorgio and Gasol, 2008) but on average presumably >20%.

One potential constraint of the BrdU method is that not all bacterial taxa may be able to take up this thymidine analog as already stated by Urbach et al. (1999). It has been shown that several bacterial isolates from soil and of marine origin affiliated to *Actinobacteria*, *Bacteroidetes*, *Gamma*- and *Alphaproteobacteria* were unable to take up BrdU and that individual uptake rates during exponential growth varied up to tenfold (Hamasaki et al., 2007; Hellman et al., 2011). On the other hand these and other studies found that the great majority of marine isolates, affiliated to *Actinobacteria*, *Gammaproteobacteria*, *Roseobacter*, and *Bacteroidetes*, is capable of taking up BrdU (Pernthaler et al., 2002b; Hamasaki et al., 2007; Mou et al., 2007). Also the major pelagic marine lineage of *Alphaproteobacteria*, the SAR11 clade, was shown to take up BrdU (Tada et al., 2011, 2013). These authors reported that BrdU-positive SAR11 cells constituted 2 to 33% of total bacterial cells detected by CARD-FISH. Hence, these data suggest, in accordance with a previous statement (Pernthaler et al., 2002b), that BrdU does not show any indication of toxicity on uptake by the great majority of marine bacteria. In the present study we detected the major and well known bacterial phylogenetic groups as BrdU-positive and thus assume that in our samples no group was specifically affected by a potential toxicity of BrdU. The low fractions of the SAR11 clade in BrdU-positive cells, i.e., a relatively low cell proliferation and physiological activity is in line with the low gene expression levels reported for the southern North Sea as compared to *Roseobacter* RCA populations (Voget et al., 2015).

In total, more than 60% of the BrdU-positive cells were covered by the probes we applied. However, the BrdU-FISH analysis showed that the composition of the DNA-proliferating cells was very different than that of the total bacterioplankton community. *Bacteroidetes* and the *Roseobacter* group dominated among DNA-proliferating cells and the SAR11 clade and *Gammaproteobacteria*, except at one station, constituted only minor proportions. These data are in line with previous reports from the western North Pacific, the subtropical Indic and Southern Ocean (Tada et al., 2011, 2013). Interestingly, the phylogenetic groups represented among BrdU-positive cells were strikingly differently correlated to the set of biological and hydrographic variables. Only the *Roseobacter* group and its RCA cluster exhibited significant and close correlations to BP, HNA, and LNA cells and Chl*a* fluorescence and *Gammaproteobacteria* to phaeopigments, POC and PON. In contrast *Bacteroidetes* did not exhibit any significant correlation to biogeochemical variables and the SAR11 clade only to LNA cells. These results suggest that the growth dynamics of the *Roseobacter* group were much more closely controlled by phytoplankton-related processes than those of the other phylogenetic groups assessed, despite its rather small proportion of the total bacterioplankton community.

Based on the fact that the proportions of cells of a *Roseobacter* and *Alteromonas* isolate increased over time preceding cell proliferation Pernthaler et al. (2002b) suggested that BrdU uptake may be a sensitive measure of the *in situ* growth potential of the targeted phylogenetic groups. Therefore we used the BrdU uptake data to make an estimate of the potential growth rate of the *Roseobacter* group and the other phylogenetic lineages assessed. The *Roseobacter* group and its RCA cluster harbored the largest fraction of BrdU-positive cells indicating that this group was the most active, i.e., cell-proliferating component of the bacterioplankton community. Assuming a linear accumulation of BrdU by the cells (Pernthaler et al., 2002b) and taking the mean proportion of 36% of BrdU-positive cells within the *Roseobacter* group and of 32% within its RCA cluster suggests a mean growth rate of the *Roseobacter* group and its RCA cluster of 1.5 and 1.3 per day, respectively. A similar calculation yields growth rates of *Gammaproteobacteria* and the SAR11 clade of 0.21 and 0.04 per day, respectively, and of *Bacteroidetes* of 1.2 per day. The growth rates of the *Roseobacter* group and its RCA cluster and of *Bacteroidetes* are far higher than the measured bulk growth rates of the entire bacterioplankton communities, whereas those of *Gammaproteobacteria* were in the range of these bulk growth rates and those of the SAR11 clade far below these rates (**Figure 4**). The Box-Whisker plot shows that the mean, median and range of the proportion of BrdU-positive cells within *Bacteroidetes* were lower than that of the *Roseobacter* group and its RCA cluster (**Figure 8**). This indicates that cell proliferation and thus growth was generally higher in the latter group and emphasizes that the *Roseobacter* group was the most active player of the bacterioplankton community in processing phytoplankton-derived organic matter despite its relatively low proportion of the total bacterioplankton community. The RCA cluster constituted at least 15 and in 65% of the samples more than 50% of the cells of the *Roseobacter* group detected by CARD-FISH as well as BrdU. These data further emphasize the great significance of this cluster as an active component of the bacterioplankton and add to previous reports in the North Sea and other pelagic marine systems (West et al., 2008; Giebel et al., 2009, 2011; Teeling et al., 2012; Wemheuer et al., 2014; Voget et al., 2015). The low proportions of the *Roseobacter* group in the CARD-FISH data may be a result of a high top-down control by grazing and viral lysis, the major mortality factors of pelagic bacteria, of the rapidly growing cells of this group (Longnecker et al., 2010; Winter et al., 2010; Chow et al., 2014).

*Bacteroidetes* and its *Polaribacter* cluster harbored almost as many BrdU-positive cells within their groups as the *Roseobacter* group but constituted a much higher fraction of total CARD-FISH and BrdU-positive cells. However, the BrdU-positive cells of this group did not exhibit a positive correlation to fluorescence or concentrations of Chl*a* or BP and only its *Polaribacter* cluster to Chl*a* concentrations. The proportions of BrdU-positive cells of *Bacteroidetes* within this group and in particular of the *Polaribacter* cluster were lower than those in the *Roseobacter* group. Estimated growth rates of *Bacteroidetes* were also slightly lower than those of the *Roseobacter* group. These findings suggest that *Bacteroidetes*, represented presumably mainly by its *Polaribacter* cluster and other *Flavobacteriaceae* (Teeling et al., 2012), were generally intensely involved in organic matter processing, but not specifically in relation to the phytoplankton blooms we encountered in early summer. Nonetheless, our findings corroborate generally the notion that *Bacteroidetes* and the *Roseobacter* group are the key phylogenetic groups involved in organic matter processing during phytoplankton blooms in pelagic marine systems in temperate to polar regions (Buchan et al., 2014). The *Roseobacter* group, despite its lower share of the total bacterioplankton community, appears to be the more active component and prone to higher mortality losses than *Bacteroidetes*.

As a conclusion the application of CARD-FISH, BrdU-FISH and assessing BP provided a detailed insight into the partitioning of the various bacterial phylogenetic groups in the total and cell-proliferating bacterioplankton communities and allowed a refined insight into the growth dynamics of distinct phylogenetic groups in the North Sea in early summer. More than a decade ago this approach has been first tested in the North Sea (Pernthaler et al., 2002b) but so far not applied to a detailed study in this dynamic coastal sea. SAR11 dominated the total but constituted only minor proportions of the cell-proliferating bacterioplankton and exhibited low growth rates. In contrast the *Roseobacter* group constituted only minor proportions of the total but, together with *Bacteroidetes*, constituted a major proportion of the cell-proliferating bacterioplankton, exhibited the highest growth rates and the closest correlation to Chl*a* fluorescence and BP. Hence, this bacterial group appeared to be a major player in processing phytoplankton derived organic matter despite its low partitioning in the total bacterioplankton community. It seemed to be controlled top down by grazing and viral lysis but the significance of these controlling factors still needs to be shown.

## Author Contributions

SB, IB, and FM carried out the field work during the cruise. IB designed the RCA probe. IB and LD performed the CARD-FISH and BrdU-FISH analyses. H-AG carried out the flow cytometric analyses of the bacterial community, FM analyzed the biogeochemical data. LD carried out the statistical data evaluation. IB, LD, and MS wrote the manuscript and all authors commented on the manuscript. MS designed the cruise as the responsible PI.

## Conflict of Interest Statement

The authors declare that the research was conducted in the absence of any commercial or financial relationships that could be construed as a potential conflict of interest.

## Footnotes

1 http://mathfish.cee.wisc.edu/index.html

2 http://www.technobiology.ch
