# Supplementary material for: Composition of Total and Cell-Proliferating Bacterioplankton Community in Early Summer in the North Sea – Roseobacters Are the Most Active Component
Source: Front Microbiol. 2017 Sep 13;8:1771. doi: 10.3389/fmicb.2017.01771 (PMC5604061; doi:10.3389/fmicb.2017.01771)

**Supplementary information to**

**Composition of total and cell-proliferating bacterioplankton community in early summer in the North Sea – roseobacters are the most active component**

Insa Bakenhus^+^, Leon Dlugosch^+^, Sara Billerbeck, Helge A. Giebel, Felix Milke and Meinhard Simon*

Institute of Chemistry and Biology of the Marine Environment,

University of Oldenburg,

26129 Oldenburg, Germany

*Corresponding author: Meinhard Simon. Email: m.simon@icbm.de

^+^These authors contributed equally to this work.

**Table S1:** Longitude, latitude, hydrographic, biological and microbial parameters assessed at stations 1 to 18 at 3 m depth during cruise HE-425 in the North Sea between 23 May and 7 June 2014. N/A: not available.

| Station | Longitude  [E] | Latitude  [N] | Temperature [°C] | Salinity | Chlorophyll *a* [µg l^-1^] | Fluorescence [V] | Phaeopigments  [µg l^-1^] | BPP C  [ng l^-1^ h^-1^)] | POC [mg l^-1^] | PON [mg l^-1^] | HNA cells  [10^5^ cells ml^-1^] | LNA cells  [10^5^ cells ml^-1^] |
| --- | --- | --- | --- | --- | --- | --- | --- | --- | --- | --- | --- | --- |
| 1 | 7.93 | 54.08 | 13.76 | 29.88 | 1.02 | 1.18 | 0.23 | 388.9 | N/A | N/A | 15.00 | 5.76 |
| 2 | 7.38 | 54.50 | 13.12 | 32.83 | 0.61 | 0.63 | 0.04 | 203.8 | 0.16 | 0.01 | 8.27 | 0.96 |
| 3 | 6.83 | 54.92 | 13.97 | 34.08 | 0.62 | 0.54 | 0 | 121.3 | 0.19 | 0 | 8.17 | 1.12 |
| 4 | 6.30 | 55.32 | 13.16 | 34.85 | 0.26 | 0.46 | 0.16 | 131.3 | 0.15 | 0 | 4.04 | 0.95 |
| 5 | 5.77 | 55.72 | 12.80 | 34.72 | 0.34 | 0.20 | 0.06 | 85.8 | 0.11 | N/A | 3.77 | 2.97 |
| 6 | 5.23 | 56.11 | 12.82 | 34.87 | 0.36 | 0.30 | 0.10 | 61.7 | 0.19 | 0.01 | 5.33 | 5.77 |
| 7 | 4.68 | 56.51 | 12.54 | 34.90 | 0.18 | 0.24 | 0.09 | 129.0 | 0.02 | 0.02 | 7.37 | 12.30 |
| 8 | 4.00 | 57.00 | 11.96 | 35.07 | 0.14 | 0.11 | 0.13 | 144.6 | 0.16 | 0 | 13.10 | 5.98 |
| 9 | 5.04 | 58.02 | 13.09 | 28.60 | 0.37 | 0.63 | 0.80 | 79.0 | 0.22 | 0.01 | 2.51 | 3.83 |
| 10 | 7.74 | 57.81 | 13.99 | 24.50 | 1.07 | 1.45 | 0.37 | 289.3 | 0.43 | 0.03 | 9.62 | 9.15 |
| 11 | 5.67 | 57.42 | 12.84 | 33.21 | 0.30 | 0.27 | 0.24 | 118.0 | 0.21 | 0 | 9.44 | 9.84 |
| 12 | 6.00 | 57.00 | 10.97 | 35.05 | 1.30 | 1.26 | 0.57 | 126.9 | 0.28 | 0.02 | 5.45 | 6.39 |
| 13 | 6.90 | 56.10 | 13.96 | 34.04 | 1.11 | 0.73 | 0.36 | 119.6 | 0.25 | 0.01 | 1.94 | 1.64 |
| 14 | 7.30 | 55.60 | 15.19 | 32.66 | 2.46 | 1.46 | 1.06 | 564.3 | 0.56 | 0.08 | 33.60 | 11.20 |
| 15 | 7.35 | 55.53 | 15.81 | 32.70 | 2.87 | 1.67 | 0.83 | 667.2 | 0.44 | 0.05 | 17.80 | 5.91 |
| 16 | 7.70 | 55.10 | 14.57 | 31.87 | 1.66 | 3.22 | 0.66 | 429.3 | 0.20 | 0.01 | 20.70 | 6.93 |
| 17 | 8.10 | 54.60 | 14.90 | 30.53 | 1.66 | 2.66 | 0.64 | 942.4 | 0.26 | 0.03 | 64.40 | 23.80 |
| 18 | 7.95 | 54.08 | 12.50 | 32.72 | 1.10 | 1.37 | 0.57 | 246.8 | 0.25 | 0.04 | 13.10 | 3.32 |

**Table S2:** ΔG° values calculated with the mathFISH software package for probes RCA826, RCA 1001, RCA 998 and RCA996 designed against 16S rRNA sequence of *Planktomarina temperata* RCA23 (GenBank Accession Number GQ369962). General analysis for *in silico* at 46°C with 1 M [Na^+^] yielded i) ΔG°1 (free energy change of hybridization assuming a linear probe structure and a fully accessible target site); ii) ΔG°2 (free energy change of folding for the probe structure); iii) ΔG°3 (accessibility of the target with respect to the secondary rRNA structure); iv) ΔG°overall (free energy change that defines the probability of probe-target hybridization at equilibrium); [FA]m (melting formamide concentration for the probe-target duplex); Hybridization Efficiency (theoretical ratio of probe-bound target molecules to all target molecules at 0% formamide).

|  | **RCA826** | **RCA1001** | **RCA998** | **RCA996** |
| --- | --- | --- | --- | --- |
| ΔG°1 [kcal/mol] | -17.5 | -18.0 | -18.7 | -21.9 |
| ΔG°2 [kcal/mol] | 1.8 | 1.2 | 0.4 | 0.4 |
| ΔG°3 [kcal/mol] | -7.1 | -9.3 | -9.0 | -9.0 |
| ΔG°overall [kcal/mol] | -10.4 | -8.6 | -9.4 | -12.6 |
| [FA]m (%) | 19.0 | 9.5 | 16.9 | 33.3 |
| Hybridization Efficiency | 0.99 | 0.86 | 0.96 | 1.00 |

**Figure S1**:

Epifluorescence micrographs *of Planktomarina temperata* RCA23 cells visualized after hybridization with probes RCA826 (5’-ATA CTT GCT GAC GTC TGG -3’), RCA1001 (5’-CAT CTC TGG TAG TAG CAC-3’), RCA998 (5’-CTC TGG TAG TAG CAC AGG-3’) and RCA996 (5’-TCT CTG GTA GTA GCA CAG GAT-3’) at 10% formamide.

**Figure S2:**

Phylogenetic tree of *Alpha*- and *Gammaproteobacteria* based on the 16S rRNA gene sequences of the Silva SSU Ref data set NR 128 (software package ARB) showing the matches and mismatches of probe RCA996 with the OTUs of the different lineages. The first value in the shaded grey areas of the subgroups gives the total number of OTUs within the corresponding lineage and the second value the number of the matches of the probe.


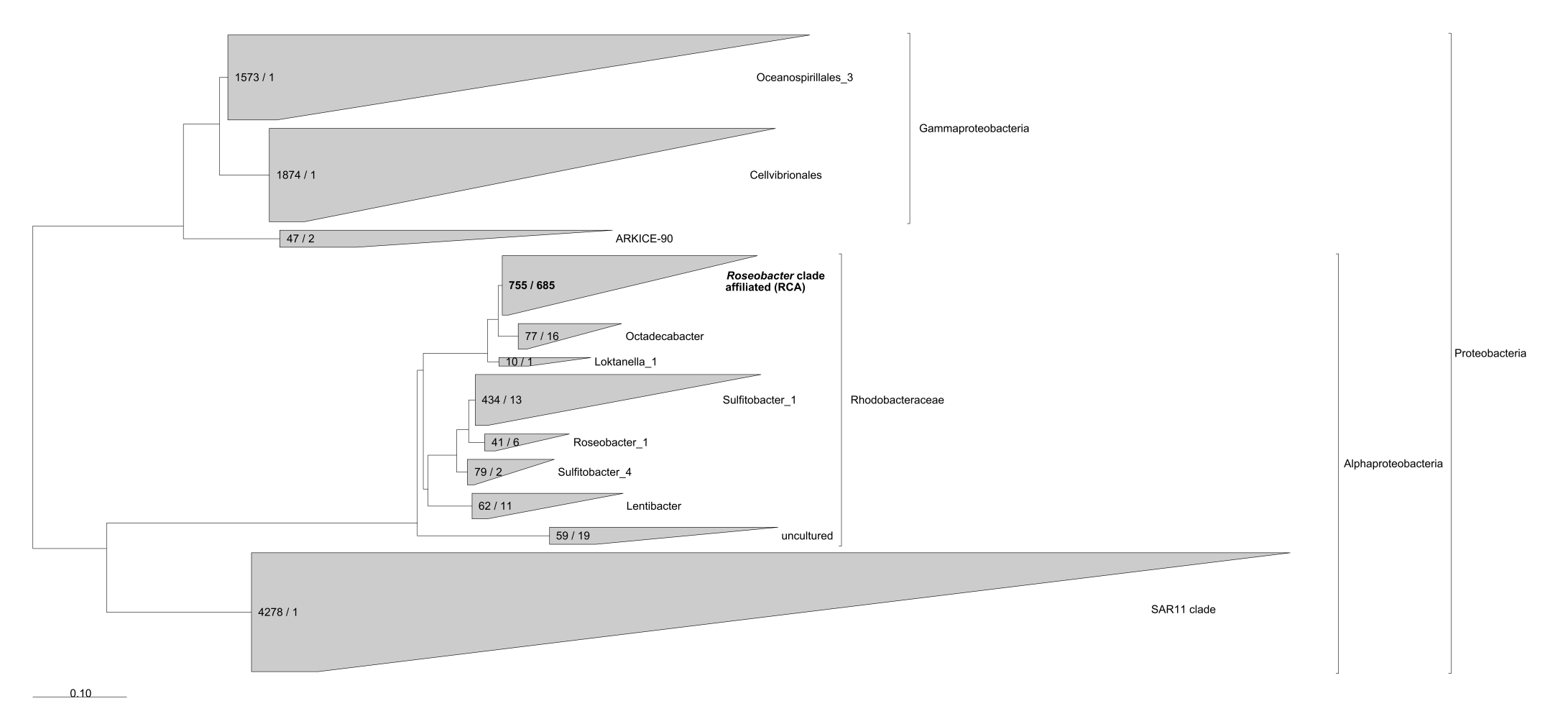

Supplement: Supplementary file 1 [file Table_1.DOCX]
